# Isolated Blowout Distal Esophageal Injury From Blunt Thoraco-Abdominal Trauma Following Airbag Deployment in an Unrestrained Driver: A Case Report

**DOI:** 10.7759/cureus.53718

**Published:** 2024-02-06

**Authors:** Hatoon Dagestani, Sahar Alomar, Doaa Alfraidy, Khaled I Twier, Ghassan Alramahi

**Affiliations:** 1 Department of Surgery, King Saud University Medical City, Riyadh, SAU; 2 Department of Trauma Surgery, King Saud University Medical City, Riyadh, SAU

**Keywords:** blowout esophageal injury, traumatic esophageal perforation, esophageal perforation, blunt esophegeal injury, blunt trauma

## Abstract

Esophageal perforation from blunt trauma is rare. It is more frequently encountered in a penetrative mechanism where the cervical esophagus is most commonly injured. Blunt esophageal injury is challenging to diagnose with nonspecific findings clinically and radiologically within trauma settings. The main factors contributing to difficulty in early recognition are its scarce occurrence combined with nonspecific manifestations clinically on patient examination and radiologically on usual trauma computed tomography with intravenous contrast. We report a case of a 15-year-old young man who sustained an isolated distal blowout esophageal perforation as a result of blunt thoraco-abdominal trauma. Despite early primary surgical repair, a leak developed later on, which was managed with stent placement. The leak and associated sepsis were resolved, with an overall status improvement and no subsequent complications. We report an unusual presentation of esophageal perforation from blunt trauma, which was promptly diagnosed and managed with multiple modalities. This case highlights the importance of early recognition and management of esophageal injury and, furthermore, the role of multiple diagnostic and therapeutic modalities that lead to a successful outcome.

## Introduction

Traumatic perforation of the esophagus due to a blunt mechanism is rarely encountered, accounting for less than 1% of all trauma cases [1]. The primary mechanism of esophageal injury is penetrating trauma with a 10:1 ratio of penetrating to blunt esophageal traumatic injuries. Gunshot wounds occur in 70-80% of penetrative cases, followed by stab injuries in 15-20% [1-3]. Diagnosis is frequently challenging with nonspecific findings clinically and radiologically. Clinical findings upon physical exam in the setting of trauma are usually not clear, with ill-defined signs and symptoms. Radiological evidence of esophageal injury includes air and fluid within the chest and abdominal cavities that could be attributed to other more common causes. A CT scan usually shows stigmata of perforation, which may include extraluminal gas locules or free fluids adjacent to the esophagus in the mediastinum or abdominal cavity, pneumomediastinum or pneumothorax, or pericardial or pleural effusions [1,3]. Prompt surgical repair is the management of choice, with the use of a buttress for repair reinforcement [2,3]. We report a case of isolated blowout esophageal perforation following blunt thoraco-abdominal trauma. This report discusses the early findings that raised suspicion of esophageal injury, initial surgical intervention, and leak management. Moreover, it emphasizes the significance of timely diagnosis and multi-management modalities that lead to patient recovery.

## Case presentation

**Figure 1:**
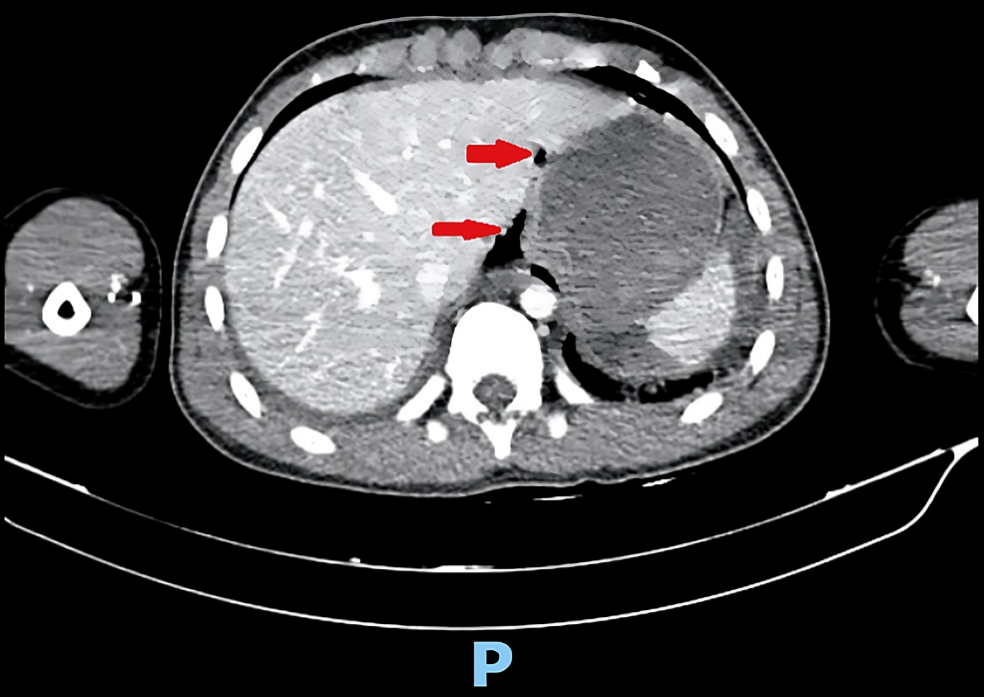
Axial view from the initial CT with IV contrast of the chest showing the esophagus surrounded by fluid and air (signs of esophageal disruption).

A 15-year-old male, medically and surgically free, was transferred to King Saud Medical City (KSMC), a level 1 trauma center, from a peripheral hospital after 5 hours of sustaining blunt thoraco-abdominal trauma. He was a victim of a front-impact motor vehicle collision (MVC) in which he was an unrestrained driver with airbag deployment. We theorize that the patient's trunk was entrapped between the driver seat and the steering wheel while the airbag was deployed, with compression from the steering wheel on the abdomen and from the airbag on the chest. This created a valve-like effect leading to sudden increased pressure in the lower esophagus, which caused his unusual injury. Upon presentation to KSMC, he was hemodynamically stable; heart rate 105, blood pressure 103/73, saturating well on room air, conscious, and oriented with a Glasgow Coma Scale of 15/15. A left intercostal tube (ICT) was in place, inserted at the previous hospital, with minimal bloody output (O/P). The abdominal exam showed localized tenderness in the epigastrium and left-side chest wall bruises; otherwise, the physical exam was unremarkable. An Extended Focused Assessment with Sonography in Trauma (E-FAST) and chest X-ray were unremarkable. He underwent CT with IV contrast (initial CT), in which CT-Chest, Abdomen, and Pelvis (CT-CAP) showed lung contusions, minimal bilateral pneumothorax, and poorly visualized distal esophagus due to adjacent cystic lesions with air-fluid levels surrounded by multiple air pockets that extended to the retro-pancreatic region (Figure 1). As the initial CT findings (pneumothorax, fluid, and air foci around the esophagus) were highly suspicious for esophageal injury, the patient was started on antibiotics and antifungals.

**Figure 2:**
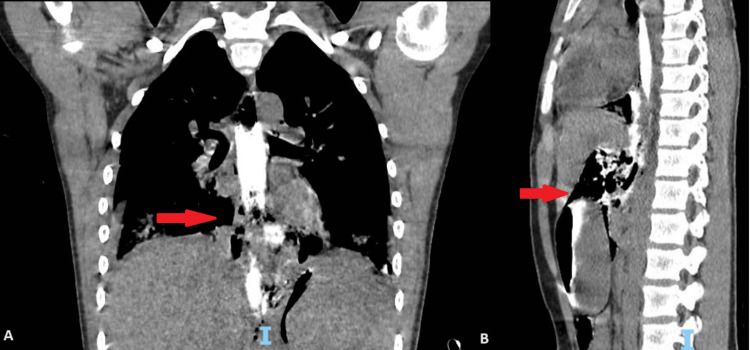
CT with oral contrast in coronal (A) and sagittal (B) views showing a contained leak in the periesophageal area.

To confirm the esophageal injury, a CT-CAP with oral contrast was performed, which showed a contained leak in the periesophageal area in continuation with the contrast extravasation in the abdomen, just abutting the stomach fundus. In addition, there were large pockets of periesophageal air foci that extended to the upper abdominal area and thorax, with contrast foci adjacent to the stomach and pancreas (Figure 2).

**Figure 3:**
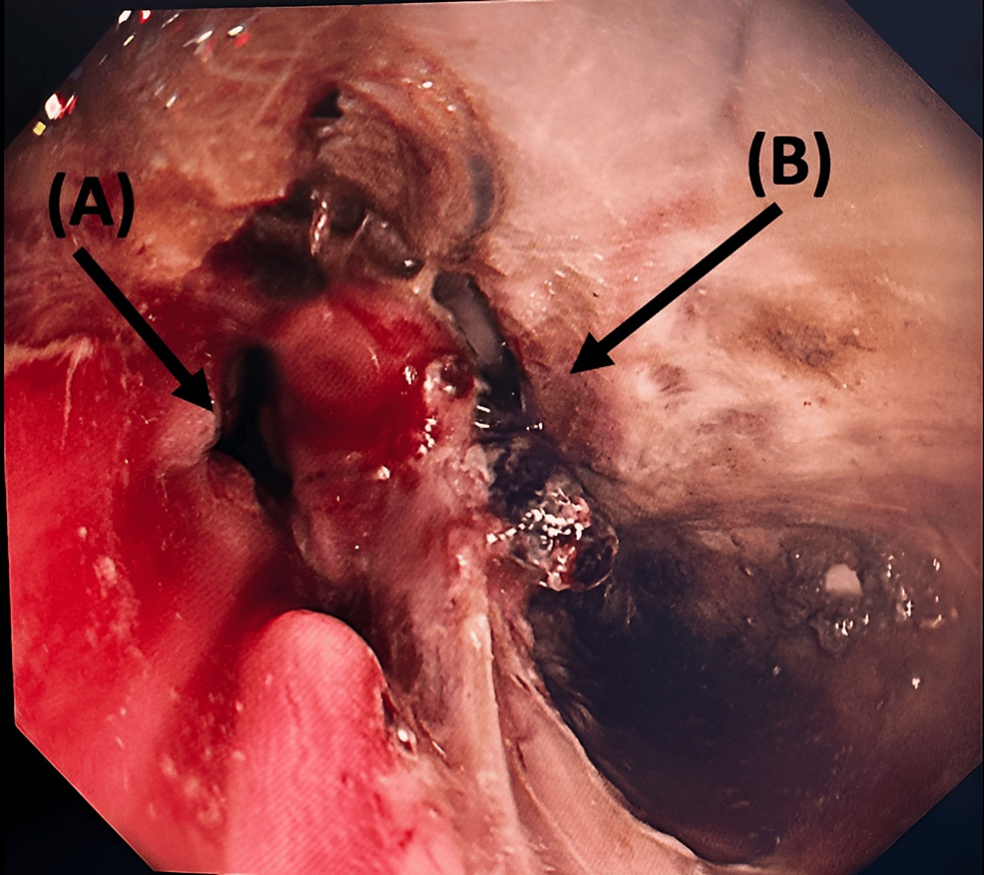
Endoscopy view: Arrow (A) shows the true esophageal lumen, and arrow (B) shows the false esophageal lumen.

**Figure 4:**
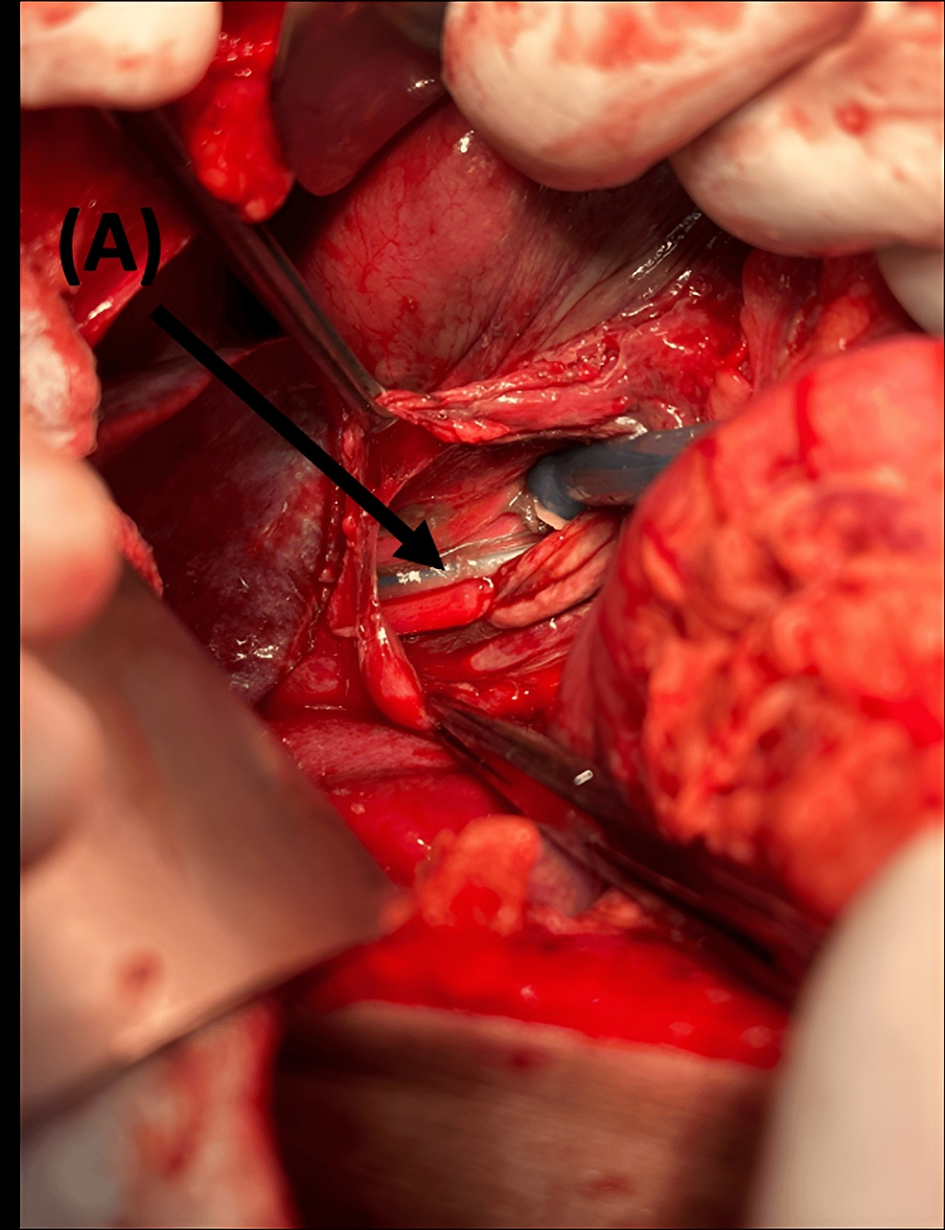
Intraoperative view of a longitudinal esophageal perforation. Arrow (A) shows the NGT inside the esophageal lumen. NGT: Nasogastric tube.

The patient was immediately shifted to the operating room (OR) for emergency surgery under general anesthesia. The procedure started with esophagogastric endoscopy by the Gastroenterology team, which identified the perforation location at the distal esophagus, proximal to the gastroesophageal junction, and to intubate the stomach (nasogastric tube (NGT) insertion) under vision (Figure 3). As the site of injury was intra-abdominal, we proceeded with exploratory laparotomy, which revealed an isolated longitudinal blowout perforation measuring 7 cm (Figure 4).

**Figure 5:**
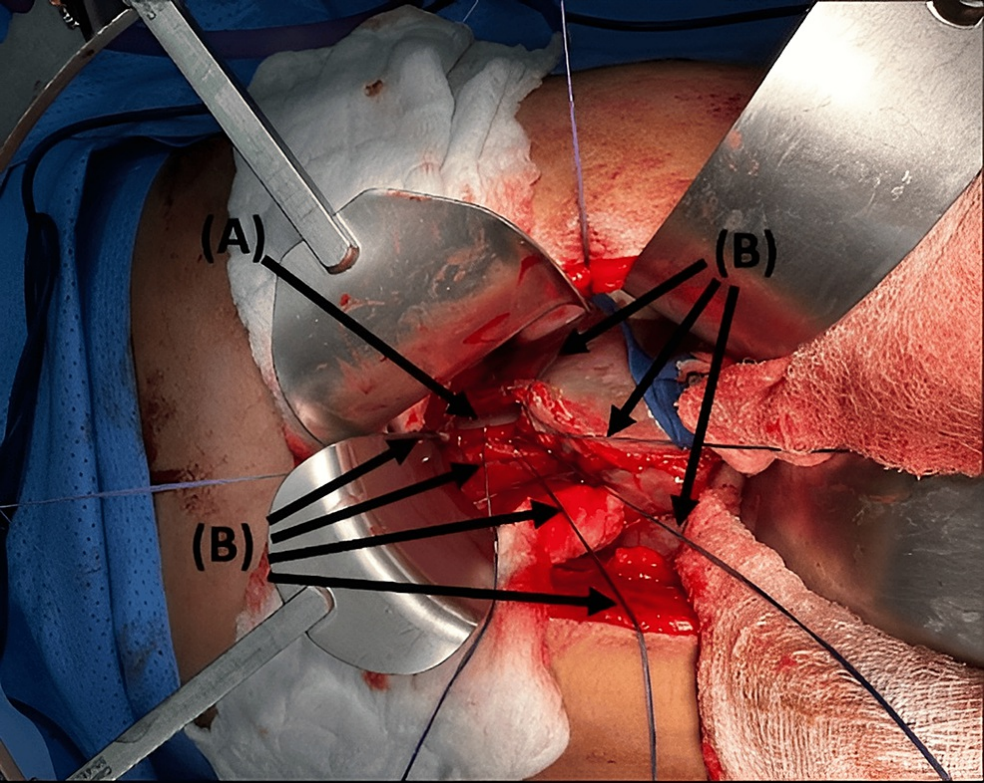
Arrow (A) shows the NGT. Arrows (B) show multiple stay sutures on the esophageal mucosal and muscular layers. NGT: Nasogastric tube.

**Figure 6:**
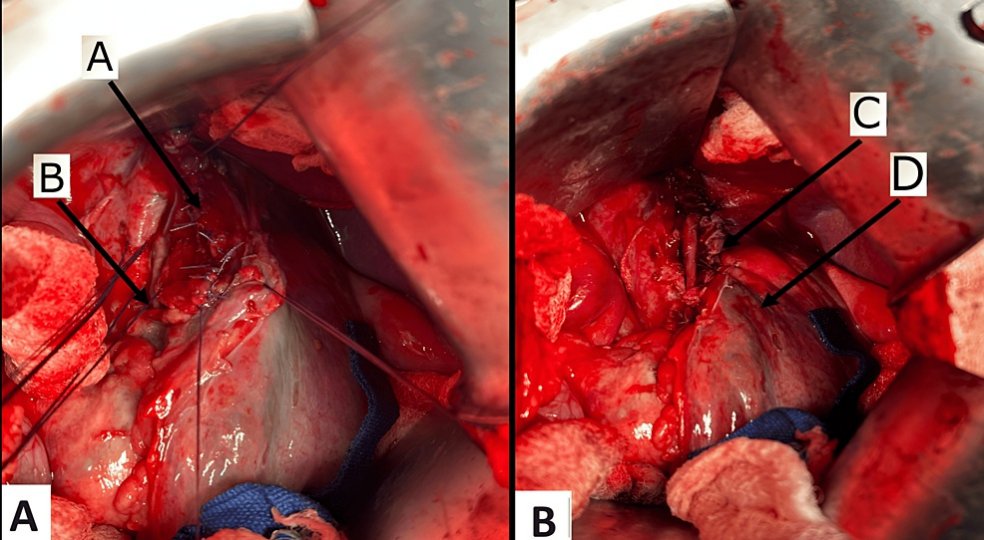
Intraoperative views: Arrow (A) shows the repaired mucosal layer; arrow (B) shows stay sutures on the esophageal muscular layer; arrow (C) shows the repaired muscle layer; arrow (D) shows the stomach.

**Figure 7:**
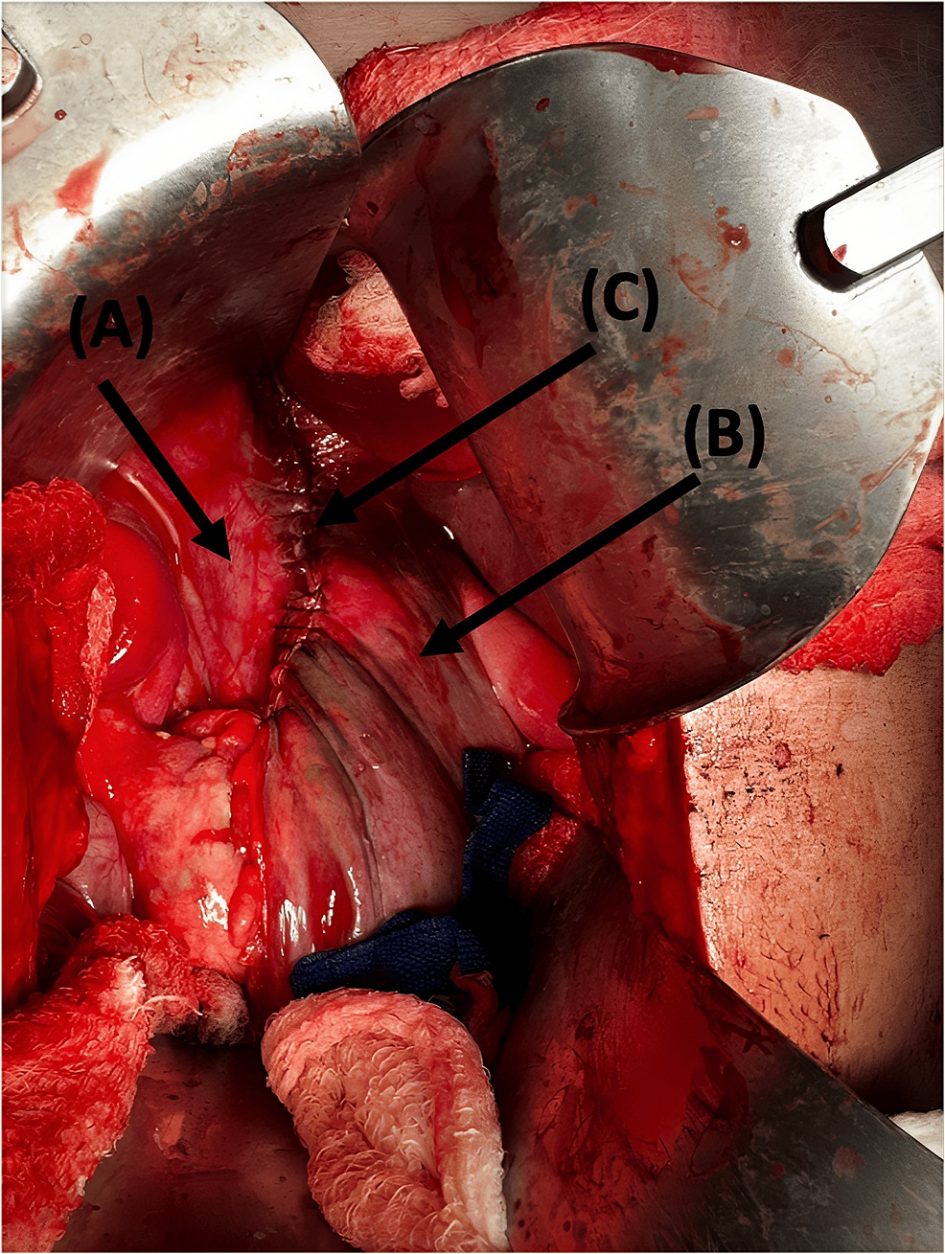
Intraoperative view: Arrow (A) shows the diaphragm, arrow (B) shows the stomach, and arrow (C) shows the stomach sutured to the diaphragm to reinforce the repair as buttressing.

Since the time of injury to the OR was within 12 hours, a decision was made to proceed with primary esophageal repair. The esophageal muscular layer was opened at the injury site inferiorly and superiorly to expose the mucosa, ensuring the extent of injury; the site looked unhealthy, for which the edges were refreshed. Muscular and mucosal esophageal layers were identified, and multiple stay sutures were placed (Figure 5). The perforation was primarily repaired in 2 layers using Vicryl 3-0 in an interrupted fashion; first, the mucosa was closed (Figure 6A), and afterward, the muscular layer was closed (Figure 6B). Finally, the repair was buttressed with the wall of the stomach, in which the stomach was sutured to the diaphragm using a running suture Vicryl 3-0 (Figure 7). Two large caliber drains were inserted into the upper abdomen bilaterally near the repair site, which were kept on the free drain, as well as a right ICT, which was connected to an underwater seal.

**Figure 8:**
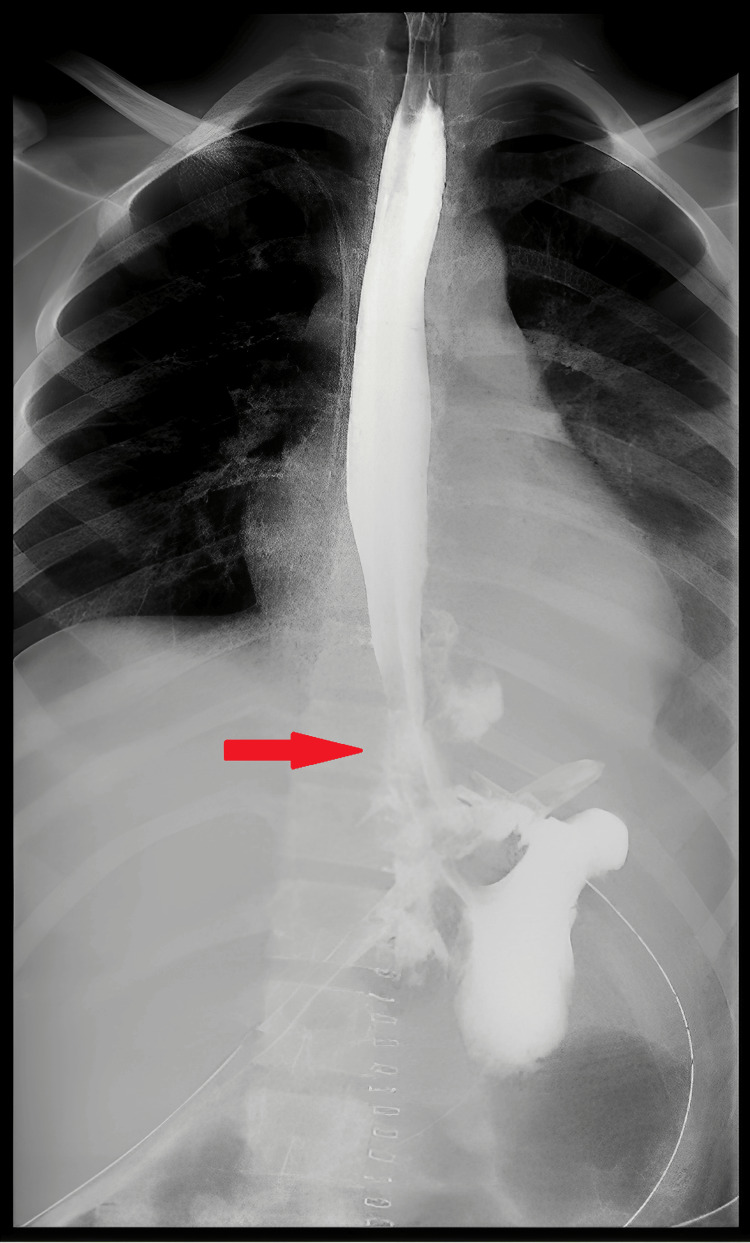
Fluoroscopy with oral contrast (Gastrografin) showing an esophageal leak without complete disruption post-repair.

Postoperatively, the patient was kept sedated on mechanical ventilation (MV) and on total parenteral nutrition (TPN). Since day 1 post-operation (post-op), the patient was continuously febrile with high-grade fever, on/off low-dose norepinephrine (NE) (0.01-0.05), with serous output (O/P) from ICTs and drains. On day 3 post-op, the right ICT was removed, and antimicrobials were upgraded. As the patient's status was not improving and in continuing sepsis, a CT-CAP with IV contrast was done on day 5 post-op. The CT showed a redemonstration of large paraesophageal air pockets and mild fluid extending to/from the upper abdominal area, in keeping with the known esophageal tear; the left ICT was removed on the same day. On day 8, purulent O/P was noticed from the right abdominal drain. Fluoroscopy with Gastrografin swallow was performed, which showed a significant leak at the repair site without complete disruption (Figure 8).

**Figure 9:**
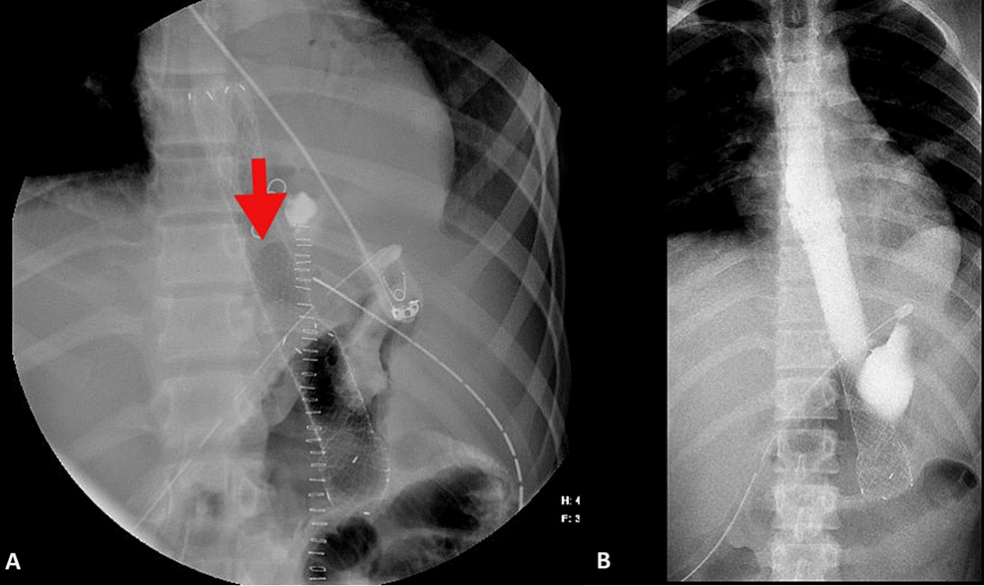
Fluoroscopy for the placement of an esophageal stent (A), which contained the leak, evident by the restriction of oral contrast within the esophageal lumen (B).

An esophageal stent was placed on day 9 (Figure 9); afterward, the patient's status started to improve; the fever subsided, fluid collection decreased in size, and he was weaned off MV and extubated on day 11 post-op. Oral feeding was started gradually and was well tolerated; then, TPN was stopped. All antimicrobials were stopped, and intra-abdominal drains were removed. The patient was discharged home two weeks after stenting in good health, to be readmitted for stent removal.

**Figure 10:**
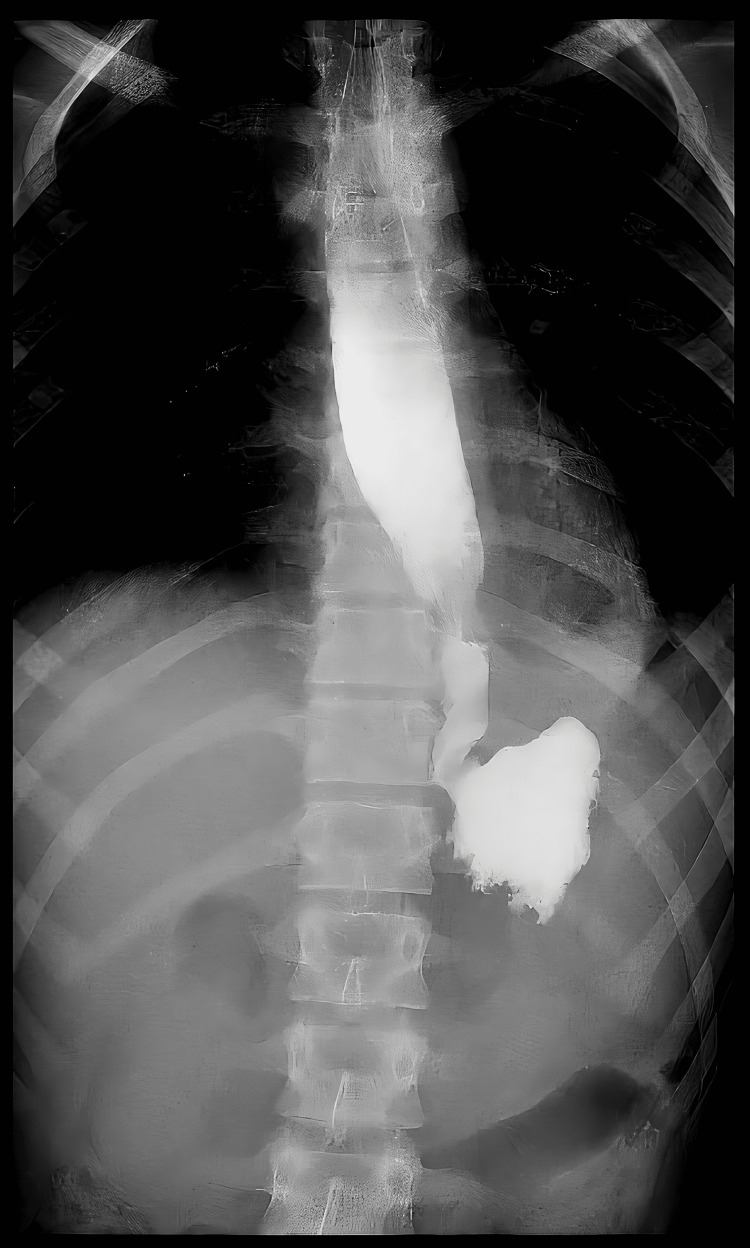
Fluoroscopy with oral contrast post-stent removal showing no leak.

The stent was removed four weeks after stenting. A follow-up fluoroscopy was done two days after removal that was unremarkable (Figure 10). The patient was discharged home in good health and followed up in the clinic one month after removal with no further complications.

## Discussion

Esophageal traumatic injuries (ETIs) are categorized into cervical, thoracic, and abdominal ETIs, following the anatomical regions of the esophagus. Cervical ETIs are the most common, followed by thoracic and then abdominal ETIs, with thoracic ETI carrying the highest morbidity and mortality, and cervical ETI carrying the lowest mortality [3]. Isolated injury to the esophagus is unusual due to its anatomical location; commonly, ETIs are seen with injuries to the airway or major vascular structures, intrathoracic or solid organs, or the vertebral column [1].

The diagnosis of ETI has the highest yield when using a collective approach, as no single examination or imaging modality is diagnostic. The presentation of esophageal perforation is nonspecific, especially in the setting of blunt trauma and associated injuries, where it might be masked by distracting injury or altered mental status. A pathognomonic triad for esophageal perforation is Mackler’s Triad: subcutaneous emphysema, chest pain, and vomiting. Commonly presenting symptoms include pain seen in 71% of cases, fever in 51%, dyspnea in 24%, and crepitus in 22% [1,4,5,6]. The most sensitive imaging modality is CT enhanced with oral contrast, which illustrates the site of the leak as well as accompanied pneumomediastinum and pleural effusion [3,7,8]. Imaging with IV contrast-enhanced CT usually shows periesophageal fluid or air, free air, and pleural effusion, which are indirect signs of perforation [7]. However, oral contrast CT is not commonly used as it requires an awake and cooperative patient [3,7].

Surgical management is the mainstay treatment approach. The operative principles for esophageal perforation include necrotic tissue debridement, tension-free defect closure with limited mobilization, buttressing with vascularized tissue, and adequate drainage [1,2,8,9]. An NGT inserted under vision and TPN in the initial phase until documented healing are recommended [6]. In 30% of patients, a leak will occur, with 40% of them requiring further intervention [9]. To the best of our knowledge, no isolated blunt esophageal rupture has been reported. In our case, the patient presented with a nonspecific exam and had suspicious findings on the initial CT. Since he was alert and oriented, we proceeded with a CT with oral contrast that showed a large distal defect. On the operative table, the site was confirmed by esophagogastroduodenoscopy (EGD), then an NGT was inserted under vision, and surgical primary repair was attempted. This was complicated by a leak, which was managed successfully by stent placement with no further complications.

## Conclusions

Traumatic blunt esophageal perforation is a rare, life-threatening condition that is challenging to diagnose and carries high morbidity and mortality. Management faces multiple difficulties, starting from the rarity of the encounter and nonspecific findings upon clinical examination or in Trauma PAN-CT that contribute to delays in diagnosis and, consequently, intervention. Surgical repair has a high rate of complications, mainly related to the nature of the esophagus. Urgent recognition and appropriate intervention are required by multidisciplinary care with multiple management modalities as needed.
